# Isolation and Identification of an Extracellular Subtilisin-Like Serine Protease Secreted by the Bat Pathogen *Pseudogymnoascus destructans*

**DOI:** 10.1371/journal.pone.0120508

**Published:** 2015-03-18

**Authors:** Evan L. Pannkuk, Thomas S. Risch, Brett J. Savary

**Affiliations:** 1 Graduate Program of Environmental Science, Arkansas State University, Jonesboro, Arkansas, United States of America; 2 Department of Biological Sciences, Arkansas State University, Jonesboro, Arkansas, United States of America; 3 Arkansas Biosciences Institute, Arkansas State University, Jonesboro, Arkansas, United States of America; IPK, GERMANY

## Abstract

White nose syndrome (WNS) is a cutaneous fungal disease of bats. WNS is responsible for unprecedented mortalities in North American cave bat populations. There have been few descriptions of enzyme activities that may function in WNS host/pathogen interactions, while no study has isolated and described secreted proteases. To address the hypothesis that *Pseudogymnoascus destructans* secretes extracellular proteases that function in wing necrosis during WNS infection, the object of this study was to culture *P*. *destructans* on various media, then isolate and structurally identify those proteases accumulated stably in the culture medium. We found a single dominant protease activity on minimal nutrient broth enriched with protein substrates, which was strongly inhibited by phenylmethylsulfonyl fluoride. This *P*. *destructans* serine protease (PdSP1) was isolated by preparative isoelectric focusing and concanavalin A lectin affinity chromatography. PdSP1 showed a molecular weight 27,900 (estimated by SDS-PAGE), broad pH optimum 6-8, and temperature optimum 60°C. Structural characterization of PdSP1 by MALDI-TOF MS, Orbitrap MS/MS, and Edman amino-terminal peptide sequencing matched it directly to a hypothetical protein accession from the sequenced *P*. *destructans* genome that is further identified as a MEROPS family S8A subtilisin-like serine peptidase. Two additional isoforms, PdSP2 and PdSP3, were identified in the *P*. *destructans* genome with 90% and 53% homology, respectively. *P*. *destructans* S8A serine proteases showed closer sequence conservation to *P*. *pannorum* and plant pathogenic fungi than to human pathogenic dermatophytes. Peptide-specific polyclonal antibodies developed from the PdSP1 sequence detected the protein in western blots. These subtilisin-like serine proteases are candidates for further functional studies in WNS host-pathogen interaction.

## Introduction

*Pseudogymnoascus* (basionym: *Geomyces*) *destructans* is the fungus responsible for white nose syndrome (WNS) in bats [1–3]. WNS has caused unprecedented mortalities in North American cave dwelling bats to the point of possible regional extinctions [4, 5]. A clinical sign in WNS-infected bats is necrosis of the wing membrane, which may lead to infarction and electrolyte imbalances [6–12]. In addition to locomotion function, bat wings play important roles in their ecology ranging from microbial protection to fecundity [13, 14]. To date, there has been no causal evidence provided for bat wing lesions. One hypothesis is that as bats exhibit frequent arousal from torpor, they “scratch” their wings to remove irritating fungal hyphae and physically damage tissues. Another hypothesis is *P*. *destructans* secretes proteases during infection, allowing mycelial penetration into underlying tissues. In addition to these possible roles in wing necrosis, immune reconstitution inflammatory syndrome (IRIS) in post-hibernating bats may exacerbate damage intensity [15].

Wing membranes consist of a thin load bearing portion and a thicker “scaffold” connective tissue trabeculae portion that serves to reinforce the wing structure [16]. The thin portion (epidermis) consists of thin epithelial cells and the keratin-rich stratum corneum. The dermis is thin and indistinguishable from the hypodermis. Elastin/collagen fibers, nerves, blood vessels, and muscle fibers are found throughout. The thicker “scaffold” region contains higher concentrations of elastin/collagen fibers and sebaceous glands. Elastin’s composition is rich in hydrophobic amino acids including glycine, valine, alanine, and proline. Structurally, collagen is a triple helix composed of amino acid triplet motifs Gly-Pro-X or Gly-X-Hyp [17]. The combination of elastin/collagen fiber network is the primary contributor to tissue elasticity; however, the contribution of each fiber type to overall elasticity is debatable [18]. The cornified cells of the stratum corneum are highly enriched in keratins, which are hydrophobic proteins with high amounts of disulfide cross-links [19]. The combination of these fibrous structural proteins creates an integumentary protective matrix aiding in innate immunity as a physical barrier to pathogenic microbes.

Fungi secrete depolymerizing enzymes to digest complex substrates in their environment for nutritional requirements. Extracellular proteases hydrolyze peptide bonds in protein catabolism to yield amino acids for assimilation [20]. Classical protease nomenclature grouped these enzymes based solely on catalytic mechanism, producing four groups: serine, metal, thiol, and acid proteases [21]. Currently, seven classes are recognized: serine, metallo-, cysteine, aspartic, threonine, glutamic, and asparagine proteases, with other proteases with unknown or mixed functions [20]. Protease classification now includes not only catalytic mechanism, but also according to the polypeptide position cleaved, primary amino acid sequence homology, and structure. Proteases are grouped into families by primary sequence homologies and further clustered in clans based on common tertiary structures [22]. Two classes frequently implicated in fungal pathogenesis include secreted metalloproteases and serine proteases [23].

Because extracellular proteases secreted by fungi may function as virulence factors, we hypothesized that *P*. *destructans* produces extracellular proteases to enable hyphal penetration into chiropteran integument. Such secreted enzymes may play a central role in pathogen establishment and bat wing necrosis during WNS. Our objective was to isolate and identify extracellularly secreted protease activities produced in *P*. *destructans* cultures. We separated a dominant protein accumulated stably in culture medium and identified it to be a subtilisin-like serine protease. This *P*. *destructans* serine protease (PdSP1) produced *in vitro* is a candidate for further functional studies in host/pathogen interactions to establish its putative role in WNS. While other studies have reported general screening for secreted enzyme activities in *P*. *destructans* cultures [24–26], this is the first isolation and description of a *P*. *destructans* serine protease and its association to a specific gene sequence.

## Materials and Methods

### Fungal Cultivation and Secreted Protein Production

*P*. *destructans* was obtained from Dr. Kevin Keel (Southeast Cooperative Wildlife Disease Study, College of Veterinary Medicine, The University of GA, USA) and cultured in the Environmental Pathogen Lab at Arkansas State University [permits: CDC (#2009–09–136), USFWS (#LE227131–0), IBC (SOP#AMP-001–111009 and SOP#AMP-002–111009)]. Stock cultures were maintained on malt agar at 8°C (Difco, Becton Dickinson, Sparks, MD, USA). To prepare inocula for protein-production cultures, *P*. *destructans* was grown on malt broth for two months. Mycelial plugs (4 of ca. 2 cm dia.) were inoculated in liquid culture media (100 ml in 2 L Fernbach flask for high surface area to volume ratio) and grown statically in the dark at 8°C. Total protease production was determined in complex culture media (Difco): tryptone peptone, brain/heart infusion, proteose peptone, tryptic soy broth (TSB), and compared with minimal nutrient broth (MNB) (NH_4_Cl 9.34 mM, NaCl 8.55 mM, K_2_HPO_4_ 1.72 mM, KH_2_PO_4_ 2.92 mM, MgCl_2_^.^6H_2_O 0.49 mM, FeSO_4_^.^7H_2_O 0.49 mM, 0.01% SDS, and 0.01% yeast extract) supplemented with 0.5% protein: gelatin (G), keratin (K), casein (C), or elastin (E).

Cultures were grown for 6–8 weeks then harvested when mycelia covered the entire liquid surface. Culture supernatants were recovered by centrifugation at 10,000 x g for 30 min, filtered through a 0.45 μm membrane, and then concentrated by lyophilization or ultrafiltration. Concentrated media were exchanged into deionized water (containing 0.02% w/v NaN_3_) with Econo-Pac 10 DG desalting columns (Biorad # 732–2010).

### Preparative Isoelectric Focusing

Preparative isoelectric focusing (IEF) was performed for six hours at 12 W with a BioRad Rotofor cell using 1% BioRad pH 3–10 ampholytes added to salt-free protein samples following manufacturer’s instructions. The pH of each fraction was recorded and adjusted to pH 7.0 with HCl/NaOH. Fractions were brought to an equal volume with ultrapure H_2_O (18 mΩ), and protein and protease activity (measured at pH 7.0) was determined for each.

### Lectin Affinity Chromatography

Glycosylated proteins were separated by lectin affinity chromatography with concanavalin A Sepharose (ConA; Sigma # C7911) following manufacturer’s recommendation. Briefly, ConA (ca. 10 ml) packed in small columns (1.5 cm ID x 12 cm) was washed with five bed volumes of binding buffer (20 mM Tris, 0.5 M NaCl, pH 8), and then sample was applied twice. Following washing the ConA column with five bed volumes of binding buffer, the glycoproteins were eluted with five bed volumes of elution buffer (20 mM α-D-methylglucoside, 0.5 M NaCl, 20 mM Tris, pH 4). The eluted fraction was neutralized with Tris buffer (pH 9). ConA samples were exchanged into ultrapure H_2_O with Bio-Rad Econopak 10DG column prior to further analysis by protein and enzyme assay or gel electrophoresis. All samples were stored with 0.02% (w/v) NaN_3_ at 4°C.

### Protein Determination and Electrophoretic Analyses

The total protein concentration was determined using Bradford’s Coomassie Brilliant Blue reagent and BSA as a standard [27]. Samples were prepared for gel electrophoresis using trichloroacetic acid (TCA) precipitation. TCA pellets were washed with ice-cold acetone, dried *in vacuo* and reconstituted in 50 μl SDS Laemmli sample buffer. Proteins in culture medium samples were separated using the BioRad Mini-PROTEAN TCX 12% pre-cast gels with the Laemmli Tris/glycine buffer system and staining with Coomassie Brilliant Blue reagent according to manufacturer’s instructions [28]. Protein size was estimated with Precision Plus Protein All Blue standards (BioRad # 161–0373) using U-SCAN-IT Graph Digitizing Software (v 5.1, Silk Scientific Corp, USA). Percentage protein composition stained bands were also estimated with U-SCAN-IT software.

Samples were desalted with Econo-Pac 10 DG desalting columns. Extracellular proteins were separated by 10% Ready Gel Zymogram Gel (gelatin; BioRad # 161–1113) following the manufacturer’s instructions. Samples were concentrated for electrophoresis with a single acetone precipitation, run in non-reducing conditions, incubated for 30 min in renaturation buffer (BioRad # 161–0765), then incubated for 16 h at room temperature in development buffer (BioRad # 161–0766). Gels were stained with 30% methanol, 10% acetic acid, 0.5% Coomassie Brilliant Blue R-250 (30 min) and destained in the methanol/acetic acid solution.

### Enzyme Activity Assays

Protease activity in culture media was initially determined with fluorescein isothiocyanate (FITC) casein according to the manufacturer’s instructions and compared to a trypsin standard (Thermo Fisher Scientific, kit # 23266). Values are expressed as units/ml, where one unit is defined as 1.0 mg / 1.0 ml trypsin standard. For determining optimal pH, protease activities were determined with azocoll (Sigma # A4341) added in 5.0 mg portions to 1.0 ml 100 mM potassium phosphate buffer, pH 7.0 at 37°C. Affinity-purified protein samples were added in 200 μl aliquots, the reaction was continued at 37°C for 30 min with gentle shaking, and absorbance was measured at 520 nm [29].

Serine protease activity of affinity-purified proteins was quantified using a peptide-*p*NA (Suc-Ala-Ala-Pro-Phe-NHPhNO_2_) colorimetric substrate (Sigma # S7388; 0.1 mM). Proteinase K (E.C. 3.4.21.64; Sigma # P6556) was used as a positive control to determine relative serine protease activity. Substrate was prepared in 0.1 M Tris-HCl, 0.01 M CaCl_2_ buffer at pH 7.5. The protein samples (100 μl) were mixed with peptide-pNA substrate (600 μl) and incubated for 10 min at room temperature, then product absorbance was determined at 410 nm and compared to a 4-nitroaniline standard curve. One unit serine protease activity was defined as 1 μmol 4-nitroaniline released per minute at 25°C.

### Biochemical Characterization

The contributions of protease classes in mixed protein samples was assessed by adding various reagents and salts followed with an activity assay with FITC casein substrate: [phenylmethylsulfonyl fluoride (PMSF, 1 mM, MeOH), iodoacetamide (IAA, 10 mM, H_2_O), sodium dodecyl sulfate (SDS, 0.5%), dithiothreitol (DTT, 0.1%), and Ca^2+^, Mg^2+^, and Zn^2+^, (all metal ions 5 mM)]. The pH optima of crude medium and affinity-purified protein were determined with azocoll as a substrate.

### *Amino*-Terminal Sequence Determination

Amino-terminal protein sequences for individual proteins blotted to PVDF membrane were determined by Edman sequencing chemistry with an AB Procise 494 instrument at the Protein Core Facility of the Iowa State University (www.protein.iastate.edu/nsequence494). Individual proteins resolved by SDS-PAGE were electroblotted to 0.45 μm Immobilon-P PVDF membrane with 10 mM CAPS, pH 11, with 10% methanol (v/v) transfer buffer for 1 hr at 150 V constant as previously described [30].

### Peptide Mass Fingerprint by MALDI-TOF MS

Structure-based protein identification was performed by peptide mass fingerprinting using trypsin digestions and separation by MALDI-TOF MS [31]. Briefly, SDS-PAGE-resolved protein bands were excised from gels, processed with 0.1% w/v Rapigest (Waters, Milford, MA, USA), and digested overnight with Trypsin-Gold (Promega, Madison, WI, USA) according to manufacturer’s instructions. Tryptic peptides were recovered and desalted with C_18_ ZipTips. Peptides were mixed with α-cyano-4-hydroxy-cinnamic acid before being spotted onto a MALDI plate. Spectra were obtained with a Waters MALDI Micro MX MS in positive reflectron mode (pulse voltage, 2,000 V; reflectron, 5,200 V; source, 15,000 V) with a 20 Hz N_2_ laser at 337 nm. Spectral data were processed with MassLynx v 4.1 and ProteinLynx Global Server 2.5 (Waters).

### *De novo* Peptide Sequencing by Orbitrap MS/MS

Sequence data from trypsin digests from affinity-purified proteins was obtained from the University of Arkansas for Medical Sciences Proteomics Core Facility (tri.uams.edu/research-resources-services-directory/core-facilities-technical-services/proteomics-core/) using a Thermo Scientific LTQ-Orbitrap Velos mass spectrometer [32]. The five most abundant peptides were selected for high-resolution tandem MS, with a 99.0% confidence protein threshold and 95.0% confidence peptide threshold.

### Bioinformatics

The peptide ion list generated by MALDI-TOF MS was used for putative fungal enzyme identification by the Mascot search engine (www.matrixscience.com). Processed spectra were submitted to MASCOT (Fixed Modification: Carbamidomethyl, Variable Modification: Oxidation, Peptide tol. ± 200 ppm) for comparison to *in silica* digested proteins. The BLASTp program at NCBI (www.ncbi.nlm.nih.gov/blast/) was used to determine gene sequence identity and correlate identity to known fungal proteases [33]. Putative enzyme function was determined with the Universal Protein Resource (UniProt; www.uniprot.org) [34]. The presence of a signal peptide was determined with the SignalP 4.1 server (www.cbs.dtu.dk/services/SignalP/). Phylogenetic analysis of *P*. *destructans* enzyme to dermatophytic and plant pathogenic fungi was performed by www.phylogeny.fr/ [35, 36]. A 3-D structure of the protein was generated by FirstGlance in Jmol (bioinformatics.org/firstglance/fgij/). Multiple sequence alignments for the three homologous PdSP proteins were performed with Clustal Omega v 1.2.0 (www.ebi.ac.uk/Tools/msa/clustalo/).

### PdSP1 antiserum and Western Blotting

Rabbit polyclonal antibodies recognizing PdSP1-specific peptide GSVDSTDTRASSSN were generated and affinity purified by the GenScript Corporation (Piscataway, NJ, USA) [37]. Peptides were selected by sequence hydrophilicity, orientation, sequence length, and homology to closely related proteins identified by a BLASTp search. Proteins were electroblotted to PVDF membrane in the manner described to prepare samples for *N*-terminal sequencing. Protein-blotted membranes were blocked with 20% nonfat dry milk and incubated in PdSP1 primary antibody (1:10,000) in Tris buffered saline (TBS) tween solution (1.5 M NaCl, 0.5 M Tris-base, 1 ml/L Tween 20, pH 7.5). Proteins were incubated in secondary goat anti-rabbit alkaline phosphatase conjugate antibody (1:3,000) in TBS-Tween and visualized with nitro-blue tetrazolium chloride and 5-bromo-4-chloro-3’-indolyphosphate *p*-toluidine salt in alkaline phosphatase buffer (150 mM NaCl, 1 mM MgCl_2_, 100 mM Tris, pH 9).

## Results

### Fungal cultivation and enzyme production

**Fig 1:**
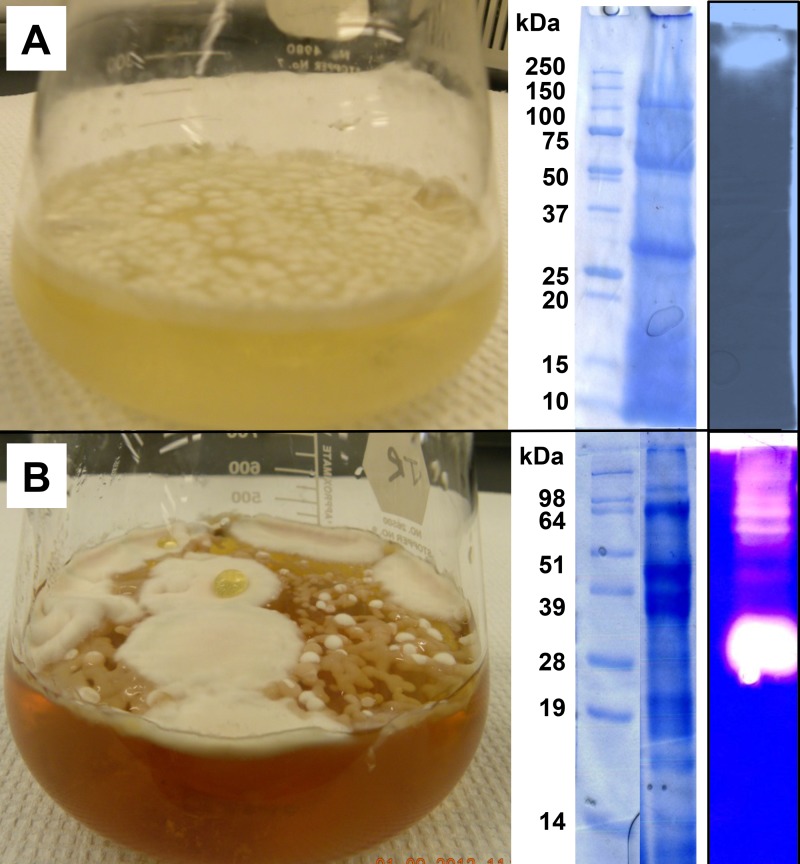
*Pseudogymnoascus destructans* growth in static liquid cultures. Left panel depicts typical culture morphology in minimal nutrient broth with gelatin (A) compared to a nutritionally complex tryptic soy broth (B). The right panel depicts a SDS-PAGE (Coomassie-Brilliant Blue G-250 stain) of extracellular proteins recovered from culture media and a native gel zymogram (casein) illustrates protease activity from total protein extract.

Representative *P*. *destructans* cultures comparing growth on MNB (minimal nutrient broth with supplemented protein, *e*.*g*., MNB+G) media, on complex fungal culture media (*e*.*g*., TSB), and extracellular protein profiles are shown in Fig. 1. More diffuse and slower hyphal growth was observed on defined MNB, requiring ~6 weeks to form a mycelial mat across the surface. Hyphal-growth occurred more quickly on complex media (~4 weeks for considerable hyphal mass), but the mycelial mass formed thick localized clumps rather than the thin mycelial mat observed on MNB surfaces. Growth on MNB resulted in consistent secreted protein profiles (observed by SDS-PAGE) regardless of protein supplement used (G, E, C, or K), while growth on complex media produced variable protein profiles. Protease zymogram screening of culture media showed two different activity profiles represented by MNB+G and TSB, which indicated differences in the dominant protease activities present. The location of protease activity at the top of the gel MNB media indicated the protein migrated into the gel poorly, whereas the dominant activity in the TSB migrated midway down the gel, indicating distinct differences in size and charge for the associated protein.

**Fig 2:**
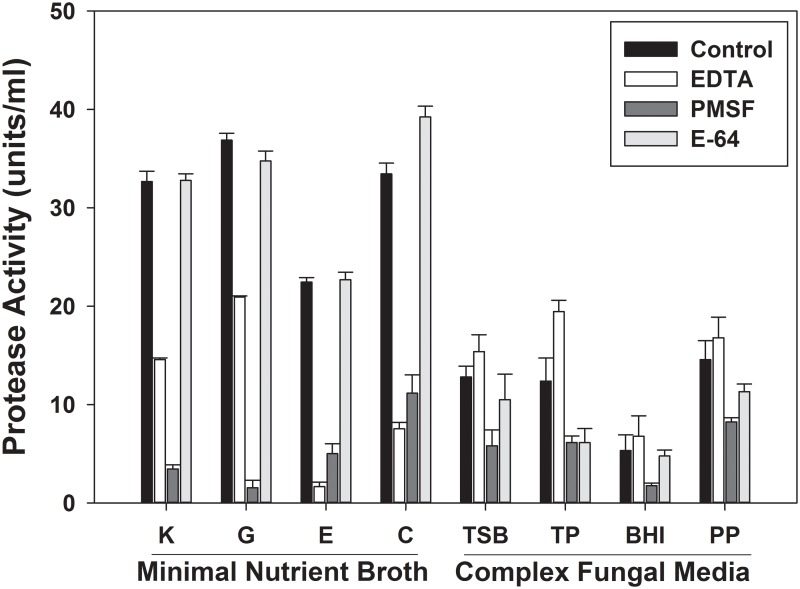
Total protease activity (units x 10^3^/ml) secreted by *P*. *destructans* grown in static liquid cultures. Various protein-supplemented minimal nutrient broth media and complex culture media were used. Controls are total activity accumulated in culture medium, and residual activity was determined following treatment with the protease inhibitors EDTA, PMSF, and E-64. Minimal nutrient broth with K, keratin, G, gelatin, E, elastin, C, casein. Complex media: TSB, tryptic soy broth, TP, tryptone peptone, BHI, brain-heart infusion, PP, proteose peptone. (Mean and standard error for triplicate samples.)

Qualitative differences were observed in total protease activity (determined with FITC casein substrate assay) secreted into culture media (Fig. 2). *P*. *destructans* grown on MNB with supplemented proteins accumulated ~3X more total protease activity compared to the complex media treatments. MNB samples treated with representative protease inhibitors (EDTA, PMSF, and E-64) showed a dramatic reduction in activity occurred with PMSF, indicating dominant serine protease activity. E-64 treatment resulted in no protease activity inhibition, indicating measurable cysteine proteases were not secreted in media tested. EDTA treatment resulted in pronounced inhibition in MNB media only. While EDTA generally inhibits metallo-proteases, it can also de-stabilize other broad class activities, including certain serine proteases. Though metallo-proteases may be secreted in MNB media, they likely represent a minor class compared to serine proteases due to the singular activity band observed by zymogram analysis (Fig. 1A). MNB+G provided the highest total protease activity yield, with nearly complete inhibition of serine protease activity with PMSF. Further experimentation to isolate serine proteases focused on culturing *P*. *destructans* with MNB+G.

### Isolation of the serine protease activity

**Fig 3:**
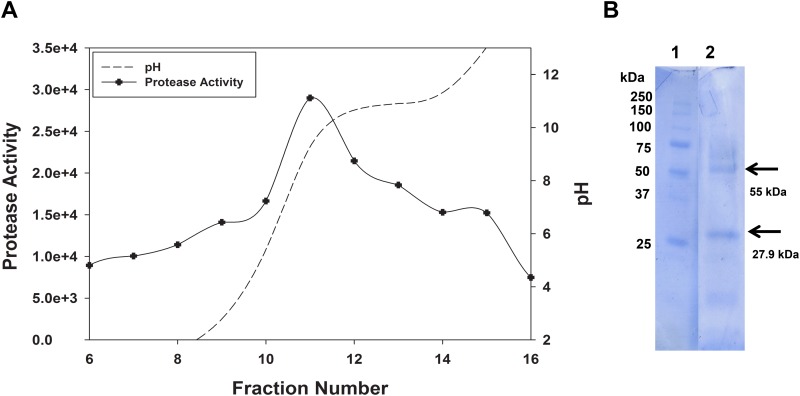
Preparative IEF separation and analysis of *P*. *destructans* extracellular proteins. Secreted proteins were recovered from MBN-G culture medium with broad-range ampholytes. Activity profile separated with broad-range ampholytes in the Rotofor cell (A). pH gradient indicated by dashed line. Protease activity assayed with FITC-casein: activity indicates fluorescence units per ml. Pooled activity peak fractions (10–13) resolved by SDS-PAGE and stained with Coomassie-brilliant blue.

While the extracellular protein profile in MNB+G culture media is simple, the considerable solution viscosity (possibly due to secreted polysaccharides) of concentrated samples was problematic for isolating the serine protease present. To address this issue, two separation methods were evaluated—preparative IEF and affinity chromatography. For the first, the concentrated enzyme solution was mixed with broad range ampholytes (pH 3–10) and fractionated in a Rotofor cell. Protease activity was resolved as a peak over fractions 10–13 (pH 7–9; Fig. 3). PMSF completely inactivated protease activity in the pooled fraction, confirming presence of the serine protease (data not shown). The SDS-PAGE profile for proteins in pooled peak fractions showed enrichment of two major bands at 27.9 kDa and 55.0 kDa in the pooled fraction (their co-isolation indicates a similar pI for both proteins).

**Fig 4:**
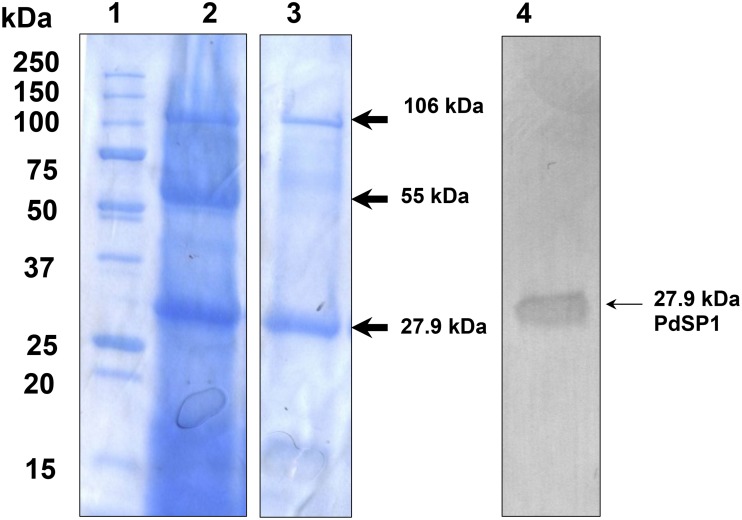
SDS-PAGE of *P*. *destructans* extracellular proteins separated by ConA lectin affinity chromatography. Lane 1, Biorad Precision Plus mass standards; lane 2, Crude enzyme substrate; lane 3, ConA elution (from non-adjacent gel lane); lane 4, western blot analysis detection in enriched protein concentrate using a PdSP1 antiserum.

Further treatment of concentrated culture medium evaluated affinity binding to benzamidine-Sepharose and ConA chromatography media. No protein or activity was adsorbed to benzamidine-Sepharose, suggesting the serine protease is not a family S1-type serine peptidase [38]. In contrast, serine protease activity in extracellular preparations bound to the ConA lectin affinity column, indicating it contains an *N*-linked glycan. SDS-PAGE of proteins eluted from ConA (Fig. 4) showed the 27.9 kDa band was greatly enriched by this separation, and the 55.0 kDa band being eliminated. The specific activity of the ConA-bound fraction was increased 33-fold, implicating the 27.9 kDa protein as the *P*. *destructans* serine protease (PdSP).

Electrophoretic and biochemical properties determined for the ConA-treated PdSP are summarized in Table 1. The enzyme showed a broad pH optimum covering pH 6 to 8. Its temperature optimum was ~60°C, suggesting modest thermal stability. Addition or DTT or SDS to substrate (casein) solution showed increased enzyme activity, while divalent cation addition did not further increase activity.

**Table 1:** Properties observed for the *Pseudogymnoascus destructans* serine protease isolated by ConA lectin affinity chromatography.

Molecular mass (SDS-PAGE)	27,900 Da
Estimated pI	pH 7 to 8
Concanavalin-A binding	Yes (*N*-linked glycoprotein)
N-terminal peptide	ALETRSGAT
pH optimum	pH 6 to 8
Temperature optimum	60°C
PMSF (1 mM)	>90% inhibition
0.1% DTT	155% activity increase
0.5% SDS	178% activity increase
CaCl_2_ (5 mM)	No change
MgCl_2_ (5 mM)	No change
ZnCl_2_ (5 mM)	No change

### MALDI-TOF MS screening to identify *P*. *destructans* proteins

There were 7 major bands observed consistently by SDS-PAGE for proteins isolated from *P*. *destructans* MNB+G culture medium (Fig. 1). All 7 proteins were excised from SDS-PAGE gels, trypsin-digested, and then analyzed by MALDI-TOF MS to establish identity based on sequence by matching tryptic peptide sets using the MASCOT search engine with the Broad Institute’s *P*. *destructans* genomic sequence database [*Geomyces destructans* Sequencing Project, Broad Institute of Harvard and MIT (www.broadinstitute.org)] and NCBI’s Reference Sequence database [39]. Tentative identifications were initially made for 5 proteins (Table 2). The largest band, 115 kD, matched to a putative catalase/peroxidase HPI. The dominant 106 kD protein, which co-separated by ConA binding, is without annotated function, but structural elements and sequence homology implicate it to be a FAD-binding oxidoreductase. A minor 84 kD protein is also putatively identified as a C-N ligase. The 55 kDa protein, enriched in the IEF peak fractions but not ConA, is putatively identified as a glycoside family 18 chitinase. The smallest protein identified, 12.5 kDa, is putatively identified as a phosphatidylglycerol / phosphatidylinositol transfer protein. The 70 kDa protein remains unidentified, but is eliminated as the serine protease since it was separated from the isolated enzyme activity by both IEF and affinity chromatography treatments.

**Table 2:** Tryptic peptide-mass fingerprint analysis by MALDI-TOF MS to identify major *P. destructans* ^1^ extracellular proteins resolved by SDS-PAGE.

Protein Mass(kDa)	Gene Identification (and Protein Accesson)	Mascot Score
115	UniProt KATG (L8FYA6_PSED2; GenBank ELR04656.1); Catalase/peroxidase HPI	87
106	UniProt GMDG_07140 (L8FX91_PSED2; GenBank ELR05098.1); Similar to fungal FAD-binding oxidoreductases	162
84	UniProt GMDG_08145 (L8G218_PSED2; GeneBankELR06854.1); Similar to fungal glutamyl-tRNA(Gln) amidotransferase subunit A	96
70	Not identified	—
55	UniProt GMDG_06569 (L8FSW0_PSED2; GenBank ELR04060); Similar to fungal GH family 18 chitinase	85
27.9	UniProt GMDG_08491 (L8G6I7_PSED2; GenBank ELR07576); S8 serine peptidase	—
12.5	UniProt GMDG_02579 (L8G2X2; GenBank ELR07487.1); Similar to fungal phospholipid transfer protein	137

^1^Gene identifications indicate ORF names from *Pseudogymnoascus destructans* ATCC MYA-4855 (strain 20631–21) genomic sequence. Identification of the 27.9 kDa protein required both MS and MS/MS data to establish unequivocal identity.

**Fig 5:**
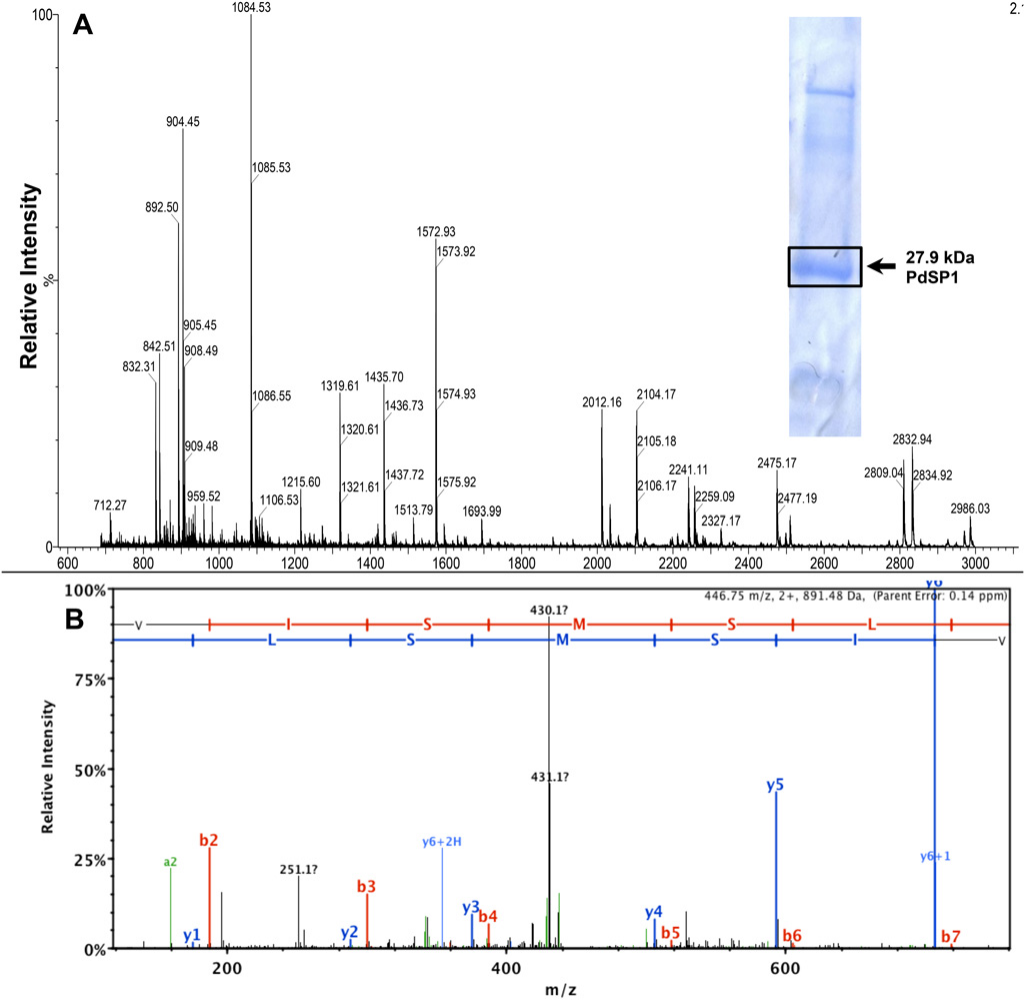
Peptide mass fingerprint and MS/MS spectra of PdSP1. MALDI-TOF MS spectrum (*m/z* 700–3000) from PdSP1 tryptic peptides (A). MS/MS spectrum with b/y-series ions from peptide ion *m/z* 908.49 (Ox+16) (SVISMSLR) (B).

The tryptic peptide mass spectrum (by MALDI-TOF MS) for the *P*. *destructans* 27.9 kDa protein is shown in Fig. 5A. While the overall sample quality appeared good, the tryptic peptide ion data was not able to provide statistically significant matching to any existing protein. Several exogenous peptides are identified in the mass spectrum—the autolytic trypsin peptide, *m/z* 842.50 and three peptides derived from ConA, *m/z* 1318.63, *m/z* 2104.06, and *m/z* 2832.34. Further analysis of tryptic peptides from the 27.9 kDa protein was performed with an Orbitrap (ThermoScientific LTQ Velos) mass spectrometer to obtain sequence data from peptide ions to establish identity as the PdSP. *De novo* sequencing was obtained for five peptide ions: *m/z* 904.46, *m/z* 892.49 (observed *m/z* 908.49 for M_Ox_+16), *m/z* 1435.70, *m/z* 2011.01, and *m/z* 3802.74, and the data are included in Table 3. A representative MS/MS spectrum is shown for peptide ion *m/z* 908.49 (SVISM_Ox_SLR) (Fig. 5B). The combined MS and MS/MS data matched unequivocally to a specific open reading frame (UniProt GMDG_08491) from the *P*. *destructans* 20631–21 genome, confirming structure with functional serine protease activity for PdSP1. The calculated tryptic peptide ions from the translated protein sequence (GenBank ELR07576.1) are summarized in Table 3.

**Table 3:** Tryptic peptide ion masses, position corresponding to the mature protein, and the corresponding amino acid sequences translated (GenBank ELR07576.1) from the *P*. *destructans* gene matched to PdSP1 (27.9 kDa protein).

Mass (M+1)	Sequence Position	PdSP1 Peptide Sequence
904.46^1^ ^,^ ^2^	6–14	SGATWGLGR
3802.74^2^	19–54	ATGSNSYIYDGSAGSGSTVYVLDTGIYIEHSEFEGR
3465.54^1^	57–91	WGANYISGSPDTDENGHGTHCAGTIAGATYGVASK
2025.00	103–123	DGFGATSATIAGINFVGQNGK
892.49^2^	127–134	SVIS***M***SLR
3952.84	135–175	GHYSAAVNSAVESTVSNGVTIVVAAGNDGDDASNYSPASAK
1435.70^2^	176–189	NAITVGSVDSTDTR
2011.01^1^ ^,^ ^2^	190–209	ASSSNYGSVVDIFAPGVNVK
2969.58^2^	223–254	SGTS***M***ATPHVAGLAAYLIGLGGLSSPAAIASK
1249.62^1^	269–281	GSVNLIAYNGNGA

^1^Four peptides are common with a homologue PdSP2 (GenBank ELR03877.1).

^2^ Peptide ions observed experimentally only.

Only peptide ions with a mass greater than *m/z* 800 are included. (Theoretical tryptic peptide ions from the propeptide and their sequences are not included.)

### Sequence analysis for the translated PdSP1 gene

**Fig 6:**
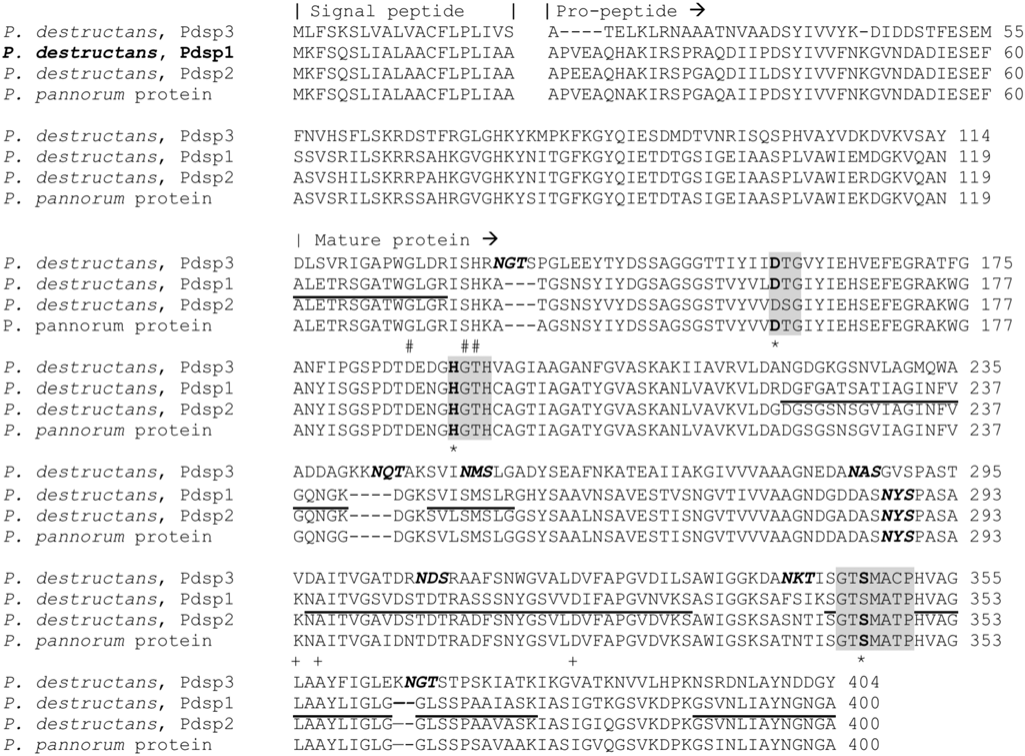
Sequence alignment of *Pseudogymnoascus spp*. Family S8 serine proteases. Sequences identified from GenBank accessions: *P*. *destructans*, PdSP3, ELR10046.1; *P*. *destructans*, PdSP1, ELR07576.1; *P*. *destructans*, PdSP2, ELR03877.1; and *P*. *pannorum* KFZ06449.1. Location of catalytic triad, D_160_, H_192_, and S_345_ (indicated by * below sequence) with conserved motifs for S8A subfamily indicated in gray boxes. Residues predicted to participate in calcium-binding are also indicated below sequences for sites C1 (+) and C2 (#). Amino acids sequences determined experimentally from PdSP1 are underlined. *N*-glycosylation sequons (N-X-S/T) are indicated in bold italics. Alignment prepared with ClustalW Omega.

The complete translated amino acid sequence for the *P*. *destructans* gene product ELR07576.1, represented by PdSP1, is shown in Fig. 6. BLASTp database searching (NCBI nrDB) indicated two conserved structural domains for PdSP1. The first includes a 99 amino acid propeptide sequence representing a MEROPS family I9 inhibitor domain, followed by the 256 amino acid sequence representing a MEROPS proteinase K-like peptidase S8 family (SB clan) domain [20]. Two serine protease homologues are present in the *P*. *destructans* genome. The first, PdSP2, shows 90% sequence identity with PdSP1, and a second homologue, PdSP3, showed a much lower 53% sequence identity. There were BLASTp hits for 43 putative S8 serine proteases in the *P*. *pannorum* genome, with sequence similarity falling between the two *P*. *destructans* homologues. The primary sequences for the three PdSP homologues from *P*. *destructans* and the highest matching homologue from *P*. *pannorum* are included in Fig. 6.

Features of PdSP primary sequences in Fig. 6 include a 20 amino acid secretory signal peptide and the location for the *N*-terminal amino acid in the mature protein (determined directly from PdSP1 by Edman sequencing chemistry; Table 1). The specific order of Asp_160_-His_192_-Ser_345_ catalytic triad (for family S8 serine endopeptidases), and the conserved motifs around them, is indicated by shading. There are two predicted calcium-binding sites in PdSP1 (C1: K_294_, A_296_, D_319_; and C2: G_132_, S_135_, H_136_). The mature PdSP1 is calculated to have a pI 7.21 and molecular weight (average mass) 27,882.70. These are consistent with the experimental results (Table 1). There is a single *N*-glycosylation sequon (N_187_-Y_188_-S_189_), which is occupied based on ConA binding, but the glycan appears to contribute little to the apparent mass by SDS-PAGE (Fig. 4). A single cysteine (C_196_) present in the mature protein sequence indicates no internal disulfide bonds in PdSP1 structure.

**Fig 7:**
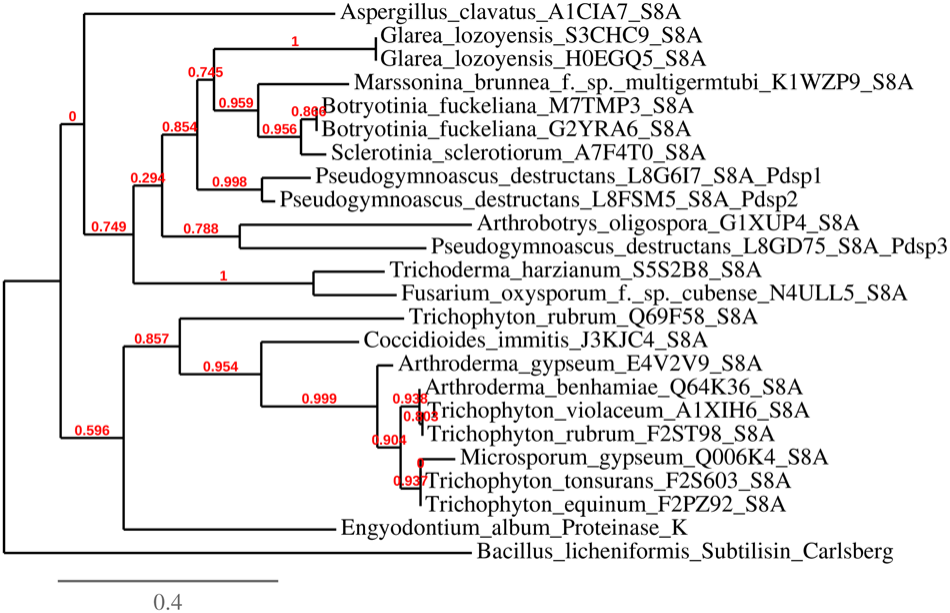
Phylogenetic tree generated from S8 serine proteases primary sequence from *P*. *destructans* and other select microbial organisms. Included are three serine proteases from *P*. *destructans*, selected serine protease sequences (S8A proteinase-K subfamily) from plant pathogenic and human dermatophytic fungi, proteinase K, Carlsberg subtilisin, and *Aspergillus cavatus* serine protease. Phylogenetic tree generated with program Phylogeny.fr (S8A accessions are listed in S1 Fig.)

A phylogenetic tree was constructed with the three *P*. *destructans* PdSP sequences and selected dermatophytic and plant pathogenic subtilisin-like serine proteases (S8A) with high sequence relationship (identified from BLASTp search), *Endgyodontium album* proteinase K, *Bacillus licheniformis* subtilisin (Carlsberg), and with *Aspergillus clavatus* serine protease chosen arbitrarily as an outgroup (Fig. 7). *P*. *pannorum* S8A serine protease sequences were not included due to the very large number of genes (43) and their close sequence conservation (as indicated by BLASTp) compared to the *P*. *destructans* PdSPs. PdSP2 and PdSP3 were 90% and 53% homologous (by sequence identity) to PdSP1, respectively. *P*. *destructans* S8 serine proteases grouped closer to plant pathogenic fungal proteases [*e*.*g*., *Botryotinia fuckeliana* (gray mold disease), *Sclerotinia sclerotiorum* (white mold), *Marssonina brunnea* (Marssonina leaf spot)] than to human pathogenic dermatophytes (*i*.*e*., *Arthroderma*, *Trichophyton*, and *Microsporum*).

### Western Blotting

The peptide GSVDSTDTRASSSN (corresponding to amino acid positions 400–413 in Fig. 6) was synthesized and used to generate a PdSP1-specific antiserum for subsequent use in western blotting detection of the protein. The resulting PdSP1 antisera showed suitable titer and sensitivity for detecting PdSP1 (27.9 kDa band) in blots from total protein isolated from *P*. *destructans* culture medium. Bovine trypsin, proteinase K, or other *P*. *destructans* proteins did not cross-react (data not shown).

## Discussion

WNS is an emergent fungal disease with devastating consequences for North American cave-dwelling bat populations, and little is known about *P*. *destructans’* ecology or relative pathogenicity [1, 2, 26]. Bats play integral roles in our ecosystem as keystone species, pollinators, and pest control [40, 41]. The unprecedented mass mortality of cave bats may lead to increases in insect pests, pesticide usage, and insect vector diseases [42]. The loss of bats and their function in natural pest control is estimated to cost at least $3.7 billion/year in damages to agriculture [43]. A clear understanding of the biochemical ecology between *P*. *destructans* and bats is paramount in determining proper control strategies to alleviate disease effects.

Extracellular proteases play roles in fungal pathogenesis and are hypothesized to function in WNS [23]. Epidermal wing necrosis observed in WNS may be partially attributed to protease activities secreted by *P*. *destructans*. Secreted serine proteases are common in saprophytic fungi and are documented in species pathogenic to plants, insects, mammals, and other fungi [44–46]. In this study we isolated a 27.9 kDa protein consistently secreted by *P*. *destructans* into liquid culture media and identified it as a family S8A subtilisin-like serine proteinase (PdSP1). We confirmed structural identification by matching peptide ion masses and *de novo* peptide sequences obtained by MS (Table 3 and Fig. 5) to the hypothetical protein (GenBank ELR07576.1), translated from the gene GMDG_08491 present in the draft genome sequence of *P*. *destructans* isolate 20631–21 (www.broadinstitute.org). The properties determined experimentally for PdSP1 (Table 1 and Fig. 5) matched values predicted for the 281 AA mature protease having a theoretical molecular weight of 27,882.7 and pI of 7.20. The nearly complete inhibition of total protease activity by PMSF indicates serine protease is the predominant protease type produced by *P*. *destructans* cultured with protein as sole nitrogen source. Subtilisin-like serine proteases are dominant secreted enzymes in fungi associated with symbiotic interactions, particularly with animals [46]. While PdSP1 is isolated as an extracellular protein produced *in vitro*, it may function as a key protease in *P*. *destructions*’ virulence.

*P*. *destructans* has three homologous genes for S8 subtilisin-like serine proteases. The translated sequence for PdSP2 (NCBI accession# ELR03877.1) exhibits high sequence identity (90%) with PdSP1, while PdSP3 (NCBI accession# ELR10046.1) retains 53% sequence identity. Considering the high sequence conservation between PdSP1 and PdSP2, there are only 4 tryptic peptides (> *m/z* 750) in common, with two of these observed experimentally (*m/z* 892.49 and 1435.70; Table 3). Four tryptic peptide ions unique to PdSP1 are observed in sample spectra, readily confirming identity matching. A recent analysis by Muszewska et al. (2011) suggested independent expansions of subtilisin-like serine proteases in fungi may be related to use of mammalian substrates, rather than pathogenicity [46]. Three isoforms in *P*. *destructans* may reflect a more obligate relationship with its host(s), whereas the entomopathogen *M*. *anisopliae* can have as many as 25 isoforms and *P*. *pannorum* has 43 S8A serine protease genes present [47], with the latter reflecting a general saprophytic lifestyle [26, 44]. The low number of additional isoforms may support previous results that *P*. *destructans* exhibits reduced saprophytic growth [26].

To facilitate preparation of PdSP1-specific antisera for future *in vivo* studies, we selected the peptide sequence GSVDSTDTRASSSN for synthesizing a peptide antigen. This peptide shows a suitable hydrophilicity profile, avoids the single *N*-glycosylation site, and appears to represent an exterior loop (determined from a 3-D model generated by FirstGlance in Jmol). We obtained a monospecific polycolonal antiserum (rabbits) from the synthetic peptide that detected the 27.9 kDa PdSP1 in western blots with good sensitivity (Fig. 4). While PdSP2 may cross-react with this antiserum, the more divergent sequence for the corresponding peptide in PdSP3 suggests it will not cross-react. Pending further characterization of the antiserum, it will prove useful service to detect the presence of PdSP1 in tissue washings or in histological sections from bats displaying WNS. Antisera may also be generated for the other two PdSP isoforms in a similar approach.

Proteases functioning as virulence factors in fungal epithelial infections may provide targets for vaccines and disease prophylactics [23]. While extracellularly-secreted proteases can be molecular biomarkers in disease processes, non-pathogenic fungi secrete many similar enzymes. Currently, the differential expression of PdSPs in *P*. *destructans* upon host tissue infection and establishment is unknown. Here, we provide two new tools for PdSP1 detection, PdSP-specific gene sequences that can be used for selecting PCR primer probes and an antiserum to directly detect the expressed product from *P*. *destructans* genes. Current evidence suggests pathogenesis is a complex process involving host, pathogen, and environmental interactions [48], hence many factors other than simply enzyme secretion contribute to disease manifestation. Furthermore, if certain enzymes involved in disease processes are inhibited, fungi may compensate by expressing alternative enzymes. *In vitro* fermentation systems provide a starting point for identifying fungal-specific extracellular enzymes. The temporal patterns of such enzymes, the presence of their active vs. precursor forms at different time points, and their secretion *in situ* within bat tissue remains an area available for future research. The availability of the *P*. *destructans* genomic sequence and application of highly accurate mass spectrometric techniques will facilitate such studies. In conclusion, we isolated and identified a dominant subtilisin-like serine protease produced *in vitro* by *P*. *destructans*, providing new tools to determine its putative function in bat wing necrosis [6], as well as for assessing expression of other PdSP isoforms during WNS.

## Supporting Information

S1 Fig Accession numbers for S8A sequences used with *P*. *destructans* proteases for preparing phylogenetic tree (Fig. 7). (DOCX)

S1 Table Summary data for total protease activity measured in culture media and response to protease inhibitor treatments (Fig. 2). (XLSX)
