# Supplementary material for: Isolation and Identification of an Extracellular Subtilisin-Like Serine Protease Secreted by the Bat Pathogen Pseudogymnoascus destructans
Source: PLoS One. 2015 Mar 18;10(3):e0120508. doi: 10.1371/journal.pone.0120508 (PMC4364704; doi:10.1371/journal.pone.0120508)
Supplement: S1 Fig — (DOCX) [file pone.0120508.s001.docx]

**S1 Figure. Accession numbers for S8A sequences used with *P. destructans* proteases for preparing phylogenetic tree (for Figure 7).**

>Pseudogymnoascus destructans L8G6I7 S8A Pdsp1

> GenBank: ELR07576.1

MKFSQSLIALAACFLPLIAAAPVEAQHAKIRSPRAQDIIPDSYIVVFNKGVNDADIESEF

SSVSRILSKRRSAHKGVGHKYNITGFKGYQIETDTGSIGEIAASPLVAWIEMDGKVQANA

LETRSGATWGLGRISHKATGSNSYIYDGSAGSGSTVYVLDTGIYIEHSEFEGRAKWGANY

ISGSPDTDENGHGTHCAGTIAGATYGVASKANLVAVKVLDRDGFGATSATIAGINFVGQN

GKDGKSVISMSLRGHYSAAVNSAVESTVSNGVTIVVAAGNDGDDASNYSPASAKNAITVG

SVDSTDTRASSSNYGSVVDIFAPGVNVKSASIGGKSAFSIKSGTSMATPHVAGLAAYLIG

LGGLSSPAAIASKIASIGTKGSVKDPKGSVNLIAYNGNGA

>Pseudogymnoascus destructans L8FSM5 S8A Pdsp2

>GenBank: ELR03877.1

MKFSQSLIALAACFLPLIAAAPEEAQHAKIRSPGAQDIILDSYIVVFNKGVNDADIESEF

ASVSHILSKRRPAHKGVGHKYNITGFKGYQIETDTGSIGEIAASPLVAWIERDGKVQANA

LETRSGATWGLGRISHKATGSNSYVYDSSAGSGSTVYVVDSGIYIEHSEFEGRAKWGANY

ISGSPDTDENGHGTHCAGTIAGATYGVASKANLVAVKVLDGDGSGSNSGVIAGINFVGQN

GKDGKSVLSMSLGGSYSAALNSAVESTISNGVTVVVAAGNDGADASNYSPASAKNAITVG

AVDSTDTRADFSNYGSVLDVFAPGVDVKSAWIGSKSASNTISGTSMATPHVAGLAAYLIG

LGGLSSPAAVASKIASIGIQGSVKDPKGSVNLIAYNGNGA

>Engyodontium album Proteinase K

>GenBank: CAA32820.1

MRLSVLLSLLPLALGAPAVEQRSEAAPLIEARGEMVANKYIVKFKEGSALSALDAAMEKI

SGKPDHVYKNVFSGFAATLDENMVRVLRAHPDVEYIEQDAVVTINAAQTNAPWGLARISS

TSPGTSTYYYDESAGQGSCVYVIDTGIEASHPEFEGRAQMVKTYYYSSRDGNGHGTHCAG

TVGSRTYGVAKKTQLFGVKVLDDNGSGQYSTIIAGMDFVASDKNNRNCPKGVVASLSLGG

GYSSSVNSAAARLQSSGVMVAVAAGNNNADARNYSPASEPSVCTVGASDRYDRRSSFSNY

GSVLDIFGPGTSILSTWIGGSTRSISGTSMATPHVAGLAAYLMTLGKTTAASACRYIADT

ANKGDLSNIPFGTVNLLAYNNYQA

>Botryotinia fuckeliana M7TMP3 S8A

>GenBank: CCD54154.1

MAGYLTVLTAFAAAFAPVFGAPAARVPHPKIKTPTTVAKDIVADSYIVVYNTDITPEVTA

SHVDFVNAIVSKRDNAVSVGANYKIHDFAGYQISADEATIVEIANKPEVAYIEKDQKVYA

SALTTQSGSTWGLGRISHRAKGTTSYIYDTTAGSGVTVYVVDTGVYAAHSQFGGRASMGA

NFVSGSANTDENGHGTHCAGTIGGSTYGVAKAAKIVGVKVLDASGSGTNSGVISGIQWVA

TNGGAKSVLSMSLGGSYSAAVNSAVTSTVAAGVTVVVAAGNDNTNAANTSPASTPNAITV

GAIDSNDARASFSNYGAVLDVFAPGVNVLSSWIGSTSATNTISGTSMATPHVAGLAAYLI

ALEGLSTPAAVVARIKALATANLITSPGSGSPNLIAYNGNGS

>Botryotinia fuckeliana G2YRA6 S8A

>GenBank: CCD54154.1

MAGYLTVLTAFAAAFAPVFGAPAARVPHPKIKTPTTVAKDIVADSYIVVYNTDITPEVTA

SHVDFVNAIVSKRDNAVSVGANYKIHDFAGYQISADEATIVEIANKPEVAYIEKDQKVYA

SALTTQSGSTWGLGRISHRAKGTTSYIYDTTAGSGVTVYVVDTGVYAAHSQFGGRASMGA

NFVSGSANTDENGHGTHCAGTIGGSTYGVAKAAKIVGVKVLDASGSGTNSGVISGIQWVA

TNGGAKSVLSMSLGGSYSAAVNSAVTSTVAAGVTVVVAAGNDNTNAANTSPASTPNAITV

GAIDSNDARASFSNYGAVLDVFAPGVNVLSSWIGSTSATNTISGTSMATPHVAGLAAYLI

ALEGLSTPAAVVARIKALATANLITSPGSGSPNLIAYNGNGS

>Bacillus licheniformis Subtilisin Carlsberg

>GenBank: CAB56500.1

MMRKKSFWLGMLTAFMLVFTMAFSDSASAAQPAKNVEKDYIVGFKSGVKTASVKKDIIKE

SGGKVDKQFRIINAAKAKLDKEALKEVKNDPDVAYVEEDHVAHALAQTVPYGIPLIKADK

VQAQGFKGANVKVAVLDTGIQASHPDLNVVGGASFVAGEAYNTDGNGHGTHVAGTVAALD

NTTGVLGVAPSVSLYAVKVLNSSGSGTYSGIVSGIEWATTNGMDVINMSLGGPSGSTAMK

QAVDNAYARGVVVVAAAGNSGSSGNTNTIGYPAKYDSVIAVGAVDSNSNRASFSSVGAEL

EVMAPGAGVYSTYPTSTYATLNGTSMASPHVAGAAALILSKHPNLSASQVRNRLSSTATY

LGSSFYYGKGLINVEAAAQ

>Sclerotinia sclerotiorum A7F4T0 S8A

>NCBI Reference Sequence: XP_001586618.1

MASYLTILTALAAVFAPVFAAPAAIPHPKIKTPTAAKEIVADSYIVVYNTDITTEVTASH

VDFVNSIVAKRDNAVSVGATYKIKDFAGYHISADEATIVEIANKPEVAYIEKDQKVYAST

LTTQSGATWGLGRISHRAKSTTSYIYDSTAGSGTTVYVVDTGVYAAHSQFGGRASMGANF

VSGSANTDENGHGTHCSGTIAGSTYGVAKAAKIVGVKVLDASGSGTTAGVISGIQWVATN

HVSKSVLSMSLGGGFSTSLNSAVTSTVASGVTVVVAAGNDNANAANTSPASTPNAITVGA

IDTNDARASFSNYGSVLDVFAPGVNVLSSWIGSTSATNTISGTSMATPHVAGLAAYLIAL

EGLSTPAAVEARIKALATSGSITNAGSGSPNLIAYNGDGA

>Marssonina brunnea f. sp. multigermtubi K1WZP9 S8A

>NCBI Reference Sequence: XP_007291353.1

MPIASEKPTLPSRYVLRAKESRMHPPRPVPLAFESSPLNNCNIGHHREKIPFEKVFRIFG

DFRVQIGSFESSSPSSISPASLDQTLLASVSVSTLVALAAVVVPAVFAAPAPAPANLKIR

NLEARDVVKDSYIVVYNPAVEADIFEASIAEVSSLISKRAVAGGIGAKYTIGDFKGYQIT

ADTATLAEIAASPDVAYIEKDQKVYASALTSQTGATWGLGRISHTLQDSTTYIYDSTAGS

GTTVYVVDTGILTTHTQFGGRATFGANYVDRSNTDGNGHGTHCAGTIAGSTYGVAKAAKL

VAVKVLAADGSGTTSGVISGIQYVGTNAPAKSVLSMSLGGTLSTALNTAVRNTIAKGVTV

VVAAGNDNKNAASFSPASEPLAITVGAIDINDNRASFSNFGALVDIFAPGVNVLSSWIGS

NSATNTISGTSMATPHVAGLAAYLIALEGLSTPAAVQARIKALGIAGKVISPGTGSPNLI

AYNGNGA

>Glarea lozoyensis S3CHC9 S8A

>NCBI Reference Sequence: XP_008088792.1

MAPYFAKLAAVAALVVPLVFGAPTPTPHLKIRNSEARDVVADSYIVVYNKDVTADVIATH

EQTVAALISKRETTGVGATFDLPGFQGYEVSADSETIAAIANSPEVAFIEKNGIMEAYDT

LAQTGAPYGLGRISHRNSSTTTYVYDSSAGTGVTIYVVDTGVYLEHEEFEGRASWGANFI

PDSPDTDEYGHGTHCAATAAGKTYGISKKAKLVAVKVLDKNGSGTFAGVISGIQWVADNA

VPNSVLTMSLGGGFSAAVNSAVAGAVSAGVTVAVAAGNSNGNISNYSPASEPSAITVGAI

DSADTRASFSNYGSLLDVWGPGVNTLSAWIGAPTATNTISGTSMATPHIAGLAAYLISLE

GLATPAAVVARIKALATSGKVKDVKSSLNLIGYNGSGL

>Glarea lozoyensis H0EGQ5 S8A

>NCBI Reference Sequence: XP_008088792.1

MAPYFAKLAAVAALVVPLVFGAPTPTPHLKIRNSEARDVVADSYIVVYNKDVTADVIATH

EQTVAALISKRETTGVGATFDLPGFQGYEVSADSETIAAIANSPEVAFIEKNGIMEAYDT

LAQTGAPYGLGRISHRNSSTTTYVYDSSAGTGVTIYVVDTGVYLEHEEFEGRASWGANFI

PDSPDTDEYGHGTHCAATAAGKTYGISKKAKLVAVKVLDKNGSGTFAGVISGIQWVADNA

VPNSVLTMSLGGGFSAAVNSAVAGAVSAGVTVAVAAGNSNGNISNYSPASEPSAITVGAI

DSADTRASFSNYGSLLDVWGPGVNTLSAWIGAPTATNTISGTSMATPHIAGLAAYLISLE

GLATPAAVVARIKALATSGKVKDVKSSLNLIGYNGSGL

>Pseudogymnoascus destructans L8GD75 S8A Pdsp3

>GenBank: ELR10046.1

MLFSKSLVALVACFLPLIVSATELKLRNAAATNVAADSYIVVYKDIDDSTFESEMFNVHS

FLSKRDSTFRGLGHKYKMPKFKGYQIESDMDTVNRISQSPHVAYVDKDVKVSAYDLSVRI

GAPWGLDRISHRNGTSPGLEEYTYDSSAGGGTTIYIIDTGVYIEHVEFEGRATFGANFIP

GSPDTDEDGHGTHVAGIAAGANFGVASKAKIIAVRVLDANGDGKGSNVLAGMQWAADDAG

KKNQTAKSVINMSLGADYSEAFNKATEAIIAKGIVVVAAAGNEDANASGVSPASTVDAIT

VGATDRNDSRAAFSNWGVALDVFAPGVDILSAWIGGKDANKTISGTSMACPHVAGLAAYF

IGLEKNGTSTPSKIATKIKGVATKNVVLHPKNSRDNLAYNDDGY

>Arthrobotrys oligospora G1XUP4 S8A

>GenBank: EGX43093.1

MHFIPLASLLPLAGLILQVAADLTVLGAGSKDKIPNSYIVVLKPSTNQAQVQEHTQRISN

YHTRRSLQERGVTTGIREQFDIQSVKGYTVECDHGTLSQILTSPEVQYVEQEGKTKVQVT

QKPSTWGLSRISWKTLPKAPYSYRYQNTWGGRGTTIYIVDSGVRISHKEFEARATWGYNA

VPDSPNTDNHGHGTHVAGTAAGKTYGVAKYARIVAVKVIGDDGTGQDSYTLAGLNYISKV

AKPGKSVVNMSIGGPKSEAVNAAVEALYKKGIVVVAAAGNENENAGLSSPASARSAITVG

ATDETDTRARFSNFGSIVDIFAPGVNILSADITSDTASRLDNGTSMASPHVAGLAAYFIS

SRTTSQSPLNIHSLFITYSQKGLVKSPGPSPNRLAYNGWDEYQPYPY

>Aspergillus clavatus A1CIA7 S8A

>NCBI Reference Sequence: XP_001272038.1

MQSIKRTLLLLGALLPAALAAPAREPHPSSNIIPGKYIITFKSGIDTAAIESHTAWASNI

HKRNLERRGLVGGEFPAGIERKFKIKDFAAYAGSFDPATIEEIRNSEDVAHVEEDQIWYL

DALTTQSGAPWGLGSISHKGQASTNYVYDTSAGAGTYAYVVDSGINVDHIEFQGRATKAY

NAVGGDHVDTLGHGTHVAGTIGGKTYGVAKQTNLLSVKVFEGRTGSTSVILDGFNWAAND

IVSKGRKGKAAINMSLGGGYSYAFNNAVESAYEQGVLSVVAAGNEGVDASNSSPASAPNA

LTVGATNKSNARASFSNYGKVLDIFAPGQDILSAWIGSTTATNTISGTSMATPHVVGLAV

YLMGLEGVSGPAAVTQRILQLATSGVISDVKGSPNKLAYNGAA

>Trichophyton violaceum A1XIH6 S8A

>UniProtKB/Swiss-Prot: A1XIH6.1

MGFITKAIPLALAAASVINGAEILETRAGVQTLADKYIVVMNDGISDKDFDSHRSWVNRN

HRRRLIRRGAKAMGGMKHTYNFPTGLKGYSGHFDEQMINEISKRADVKYIERDARVQINA

IEQQDNVPSWGLARVGSKEPGGTTYYYDSTAGEGSTAYVIDTGTDIQHEEFEGRATWGAN

FVDDMDMDCNGHGTHVSGTIGGKTFGVAKKSNVVAVKVLDCSGSGSNSGVIMGMEWATKD

AQQKGADKAVANMSLGGAFSQASNDAAAAIAKGGVFLAVAAGNDNVDAADSSPASEPSIC

TIAASTEQDSKADFSNFGQVVDVYAPGDSITSAKPGGGSQVLSGTSMATPHVAGLGAYLI

GLGKGGGPGLCDTIKQMAIDVIQNPGASTTSKLINNGSGM

>Trichophyton rubrum F2ST98 S8A

>NCBI Reference Sequence: XP_003233827.1

MGFITKAIPLALAAASVINGAEILETRAGVQTLADKYIVVMNDGISDKDFDSHRSWVNRN

HRRRLIRRGAKAMGGMKHTYNFPTGLKGYSGHFDEQMINEISKRADVKYIERDARVQINA

IEQQDNVPSWGLARVGSKEPGGTTYYYDSTAGEGSTAYVIDTGTDIQHEEFEGRATWGAN

FVDDMDMDCNGHGTHVSGTIGGKTFGVAKKSNVVAVKVLDCNGSGSNSGVIMGMEWATKD

AQQKGADKAVANMSLGGAFSQASNDAAAAIAKGGVFLAVAAGNDNVDAADSSPASEPSIC

TIAASTEQDSKADFSNFGQVVDVYAPGDSITSAKPGGGSQVLSGTSMATPHVAGLGAYLI

GLGKGGGPGLCDTIKQMAIDVIQNPGASTTSKLINNGSGM

>Arthroderma benhamiae Q64K36 S8A

>UniProtKB/Swiss-Prot: Q64K36.1

MGFITKAIPLALAAASVINGAEIMETRAGVQTLADKYIVVMNDGMTDKDFDSHRSWVNRT

HRRRLIRRGAKAMGGMKHTYRFPTGLKGYSGHFDEQMINEISKRADVKYIERDARVQINA

IEQQDNVPSWGLARVGSKEPGGTTYYYDGTAGEGSTAYVIDTGTDIQHEEFEGRATWGAN

FVDDMDMDCNGHGTHVSGTIGGKTFGVAKKSNVVAVKVLDCNGSGSNSGVIMGMEWATKD

AQQKGADKAVANMSLGGAFSQASNDAAAAIAKGGVFLAVAAGNDNVDAADSSPASEPSIC

TVAASTEQDSKADFSNFGQVVDVYAPGDSITSAKPGGGSQVLSGTSMATPHVAGLGAYLI

GLGKGGGPGLCDTIKQTAIDVIQNPGASTTSKLINNGSGM

>Trichophyton tonsurans F2S603 S8A

>UniProtKB/Swiss-Prot: A1XIH4.1

MGFITKAIPLALAAASVINGAEILETRAGVQTLADKYIVVMNDGMSDKDFDSHRSWVNRT

HRRRLIRRGAKAMGGMKYTYNFPTGLKGYSGHFDEQMIKEISKRADVKYIERDARVQINA

IEQQDNVPSWGLARVGSREPGGTTYYYDSTAGEGTTAYIIDTGTDIQHEEFDGGRATWGE

NFVDDMDMDCNGHGTHVSGTVGGRTFGVAKKSNIVAVKVLDCNGSGSNSGVIMGMQWATE

DAQSKGADKAVVNMSLGGAFSQTSNDAAKAIAEGGVFLAVAAGNDNVDAAEASPASEPSI

CTVAASTEQDGKADFSNFGQVVDVYAPGDGITSAKPGGGSQVLSGTSMASPHVAGLAAYL

IGLGKGGGPQLCDTIKQMAIDVIQNPGSSTTSKLINNGSGM

>Trichophyton equinum F2PZ92 S8A

>UniProtKB/Swiss-Prot: A1XIH3.1

MGFITKAIPLALAAASVINGAEILETRAGVQTLADKYIVVMNDGMSDKDFDSHRSWVNRT

HRRRLIRRGAKAMGGMKYTYNFPTGLKGYSGHFDEQMIKEISKRADVKYIERDARVQINA

IEQQDNVPSWGLARVGSREPGGTTYYYDSTAGEGTTAYIIDTGTDIQHEEFDGGRATWGE

NFADDMDMDCNGHGTHVSGTVGGRTFGVAKKSNIVAVKVLDCNGSGSNSGVIMGMQWATE

DAQSKGADKAVVNMSLGGAFSQTSNDAAKAIAEGGVFLAVAAGNDNVDAAEASPASEPSI

CTVAASTEQDGKADFSNFGQVVDVYAPGDGITSAKPGGGSQVLSGTSMASPHVAGLAAYL

IGLGKGGGPQLCDTIKQMAIDVIQNPGSSTTSKLINNGSGM

>Arthroderma gypseum E4V2V9 S8A

>UniProtKB/Swiss-Prot: E4V2V9.1

MGFITKAIPLALAAASVINGAEILETRAGVQTLADKYIVVMNDGMTDKDFDSHRSWVNRT

HRRRLVRRGAKAMTGMKHTYRFPTGMKGYSGHFDEQMINEIAKRADVKYIERDARVQINA

IEMQDNVPSWGLARVGSKEPGGTTYYYDSSAGQGVTAYVIDTGTDIKHEEFSGRATWGGN

FVDDIDMDCNGHGTHVSGTVAGTKFGVAKKANVVGVKVLDCDGSGSNSGVIMGMEFATND

AKKKGAGKAVANMSLGGAFSQASNDAAAAIAQGGVFLAVAAGNDNVDAAMASPASEPSIC

TVAASTEQDGKASFSNYGQVVDVYAPGDGITSAKPGGGSQVLSGTSMASPHVAGLAAYLI

GTGKSGGPQLCDTIKNMAIDVITNPGAGTTGKLINNGSGK

>Microsporum gypseum Q006K4 S8A

>GenBank: ABJ51910.1

NGAEILETRAGVQTLADKYIVVMNDGMSDKDFDSHRSWVNRTHRRRLIRRGAKAMGGMKY

TYNFPTGLKGYSGHFDEQMIKEISKRADVKYIERDARVQINAIEQQDNVPSWGLARVGSQ

EPGGTTYYYDSTAGEGTTAYIIDTGTDIQHEEFDGGRATWGENFVDDMDMDCNGHGTHVS

GTVGGRTFGVAKKSNIVAVKVLDCNGSGSNSGVIMGMQWATEDAQSKGADTSVVNMSHRV

AFSQTSNDAAKAIAEGGVLLALSSGQDNVDVAEASPASELSICTFAASTEQDGKADFSNF

GQVVDVYAAVDGITSDKPGGGSQVQSGTSKASP

>Coccidioides immitis J3KJC4 S8A

>NCBI Reference Sequence: XP_001247623.1

MVFLGKILPLALAALSVNGAEILSAPGAENIPNGYIVVMKEGTSTQDFDAHREWVASVHH

ERLARRGSTNVGGMRHTYNFNQGFMGYAGTFDEETIQEIANRDDVAYIERDQIMKASAIQ

TQRNVPSWGLARVSSRQPGGRDYSYDSTAGQGVTAYIIDTGIDIRHTDFGGRAVWGTNTV

DRRNEDCNGHGTHVAGTTGGTSFGVAKRARLVAVKVLDCNGSGSNSAVIAGMQWAMQHAS

QNDPRRAVANMSLGGGYSQATNQAAAAIVRAGIFLAVAAGNDNRDARSFSPASEPTVCTA

AASHVRDGKASFSNWGQLVDVYAPGQDIISARPGGGSRSLSGTSMASPHVCGLGAYLIGL

GRGSGGGLCDTIKRMALPVISNPGSGTTNRLINNGVSQ

>Trichoderma harzianum S5S2B8 S8A

>GenBank: AGS13697.1

MAPFNTMLAYLLMVVTPLAAAMPFHNRHSLSPQTHQHSALNMTHLMSVVINPHAKNIIAG

RYIVVYNNTFGDAAISAKQAQFAAAIAKRNLGKRDGLGNLLSTEIHSFKLHTWRAMALDA

DNDMIQSIFDSEEVAYIEADTKVQNTALVAQTNATPGLIRLSNKDVGGQNYVFDTSAASG

MTAYVVDTGIRVTHTEFEGRATFGANFVNTNDTDENGHGSHVAGTIGGATFGVAKNIKLV

AVKVLDASGSGSNSGVLNGMQFVINDVQTKKLAGKAVMNMSLGGSFSTAVNNAITALANA

GIVPVVAAGNENQDTANTSPGSAPQAITVGAIDATNDVRASFSNFGAGVDIYAPGVNVLS

VGIKSDIDTATLSGTSMATPHVAGLAAYLMALEGVTNVDDVSNLIKNLATKTGATVQQNV

AGTTDLIANNANL

>Fusarium oxysporum f. sp. cubense N4ULL5 S8A

>GenBank: EGU84012.1

MALLRTFLASLLVAAPFAAAAPIDVAETLGDMADSIGGGLGGEADKKMGISAFLRPMLKN

PDALNVIPNRYIVVYNDTFDDDTISAKEASFAAAIKKRNLNKRSSIGKAMSTSIQSFRMN

KWRAMSLDADDLMVQDLWNSDEVAYIEADTKVQLNAAIAQVNAPPGLDRLSHAKVNQDTY

VFDDSAGEGITAYVVDTGIKIDHSEFEGRATFGANFINNVDDDENGHGSHVAGTIGGATF

GVAKKVDLVAVKVLDASGGGSNSGVLQGMQFVIDDAKKKNRVGKAVMNMSLGGDFSQAIN

RAIEALFKAGIVPVVAAGNENRETALTSPGSAPNAITVGAIDATTDERADFSNFGPEVDV

YAPGVNVLSVGIKSNTDTATLSGTSMASPHVAGLAAYLMGFQQLDGPAQVASLIKSLAGQ

TGAKVQNNVQGTTDSIANNGNQ

>Trichophyton rubrum Q69F58 S8A

>NCBI Reference Sequence: XP_003236355.1

MGVFRFISISLAAVSAANAAQILSMPHAQTVPNSYIVMMKDDTSDDDFKHHQSWLQSTHT

HNITRRATIQNAGMRHKYNFNKMKGYSGIFDDETIKDIAKDPKVMFVEPDTIVSVHGKVE

QSNVPSWGLARISNPQPGADSYTYDSSAGEGITVYSVDTGVDVNHEDFEGRAIWGSNQVN

DGDDRDGSGHGTHTSGTMVGKMYGIAKKAKLVAVKVLGNDGSGPTSGIVAGINWSVEHAR

QNGGTKKAVMNMSLGGSSSSALNRAAAQAVEQGMFLSVAAGNDNQDAQSSSPASEPSVCT

VGSSAEDDSRSSFSNWGPAIDIFAPGSNIVSARPGGGSQSMSGTSMAAPHVAGLAAYLMA

LEGISGGAVCDRLKELGTSSITDAGPGTPTNVLINNGGAKGGQPNPNPAPAPSPSQPSEP

QQPTPSQPGQPGEPFPGEPFPGEPFPGQPFPGESAPAPAPAPMPPTPQHPHTPYPGGDNF

DFDSFWKKYFGGEHWRKMFSSFWN
